# P-1153. Characteristics of Invasive Group A Streptococcal Infections (iGAS) in Children during the COVID-19 Pandemic

**DOI:** 10.1093/ofid/ofae631.1339

**Published:** 2025-01-29

**Authors:** Emma Granberry, Swetha Pinninti, Suresh Boppana

**Affiliations:** UAB Heersink School of Medicine, Montgomery, Alabama; Heersink School of Medicine/University of Alabama at Birmingham, Birmingham, Alabama; University of Alabama at Birmingham, Birmingham, Alabama

## Abstract

**Background:**

Group A Streptococcus (GAS)/*Streptococcus pyogenes* is the most common cause of bacterial pharyngitis in all age groups and can also cause invasive GAS (iGAS) infections such as pneumonia, toxic shock syndrome, necrotizing fasciitis, and osteoarticular infections ± bacteremia. In the fall of 2022, the Centers for Disease Control (CDC) issued a health advisory documenting an increase in pediatric iGAS, following discontinuation of respiratory mitigation strategies (masking) during the COVID-19 pandemic. The primary objective of this study is to compare the incidence and severity of iGAS infections before and during the COVID-19 pandemic.

**Figure 1:**
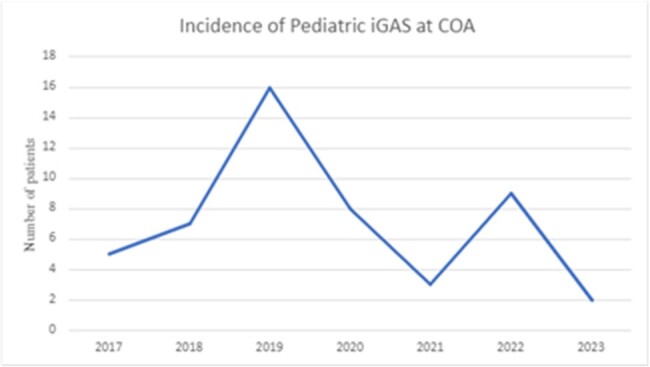
Incidence of Pediatric iGAS at Children’s of Alabama

**Methods:**

Isolation of GAS from a sterile site by culture or molecular methods was considered as iGAS. After review of electronic medical records (EMR), we identified 50 children < 18 years of age who were admitted to Children’s of Alabama (COA) between 2017 and the first quarter of 2023 with iGAS were identified. The cohort was divided into 2 groups: 1) pre-COVID-19 (Jan 2017 – Feb 2020) and 2) COVID-19 (March 2020 – Feb 2023). Demographic, clinical, and laboratory characteristics were abstracted and compared between the groups. Continuous variables were compared by the Mann-Whitney test, and Chi-square/Fisher’s exact test was used to compare categorical variables. P value < 0.05 was considered statistically significant.

**Table 1:**
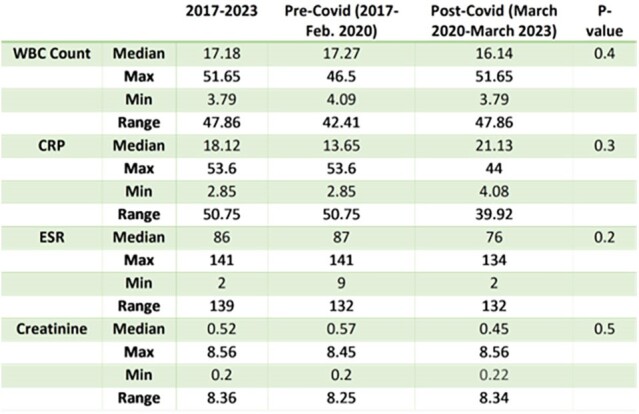
Comparison of Laboratory Parameters between Groups

**Results:**

Incidence of iGAS infection at COA plummeted during the COVID-19 pandemic from a peak of 16 cases in 2019 to 3 in 2021, followed by an increase in incidence but still lower than the pre-pandemic levels after the discontinuation of respiratory mitigation strategies (Figure 1). No significant difference between the two groups was observed for severity of infection (p = 1) and laboratory markers (Table 1).

**Conclusion:**

In this single center, retrospective study of pediatric iGAS infections, the number of children with iGAS infections decreased dramatically in 2020 and 2021 with an increase in 2022. We did not observe the increase in nationwide iGAS cases reported by CDC. Also, we did not observe significant differences in the severity of iGAS infections despite the rise in cases after the discontinuation of pandemic-era masking mandates.

**Disclosures:**

**Swetha Pinninti, MD**, Moderna: Grant/Research Support|Pfizer: Grant/Research Support **Suresh Boppana, MD**, GSK: Advisor/Consultant|Merck: Grant/Research Support|Pfizer: Grant/Research Support

